# Clinical and pathological features of *BRCA1*/*2* tumors in a sample of high-risk Moroccan breast cancer patients

**DOI:** 10.1186/s13104-016-2057-8

**Published:** 2016-04-29

**Authors:** Hassan Jouhadi, Amal Tazzite, Houssine Azeddoug, Asmâa Naim, Sellama Nadifi, Abdellatif Benider

**Affiliations:** Mohammed VI Cancer Treatment Center, Ibn Rochd University Hospital, Casablanca, Morocco; Genetics and Molecular Pathology Laboratory, Medical School of Casablanca, Hassan II University, 19 Rue Tarik Ibnou Ziad, B.P. 9154, Casablanca, Morocco; Laboratory of Biochemistry and Molecular Biology, Faculty of Sciences, Hassan II University, Casablanca, Morocco

**Keywords:** Breast cancer, *BRCA1*, *BRCA2*, Clinicopathological, Characteristics, Morocco

## Abstract

**Background:**

*BRCA1* and *BRCA2* genes explain a large part of hereditary breast cancer. Several studies have shown that *BRCA1* and *BRCA2* tumors exhibit some specific morphological and immunohistochemical characteristics. The aim of our study is to compare the clinicopathological characteristics between Moroccan breast cancers associated or not with *BRCA1* and *BRCA2* mutations. Previously, we had identified 11 *BRCA* carriers in a series of 40 selected breast cancer patients at increased risk for carrying a mutation in the *BRCA1* and *BRCA2* genes. The clinical and pathological features of patients carrying *BRCA1* or *BRCA2* mutation (n = 11) were evaluated and compared to those of non-mutated patients (n = 29).

**Results:**

In comparison with non carriers, women with *BRCA1*/*2* mutation present younger mean age at diagnosis (37.90 vs. 44.48 years, p = 0.05), younger mean age of 1st menarche (13.08 vs. 14.24 years, p = 0.05) and shorter duration of breastfeeding (8.71 vs. 19.35 months, p = 0.05). Moreover, 63.6 and 62.5 % of *BRCA1*/*2* carriers present SBR grade III and triple negative tumors respectively (p = 0.02).

**Conclusions:**

In this first Moroccan study comparing clinical and pathological characteristics of women carrying or not *BRCA1*/*2* mutation, patients with *BRCA* mutation tend to develop early breast cancer with high-grade and triple negative tumors. However, further large scale research including more data is needed to better characterize *BRCA1*/*2* cases and to evaluate the survival rate associated with these mutations in our population tumors. Moreover, it would be more interesting to study women with *BRCA1* and *BRCA2* mutations separately in order to determine if they exhibit distinct characteristics.

## Background

Breast cancer can occur in sporadic or hereditary forms. In the case of hereditary forms a germline mutation in a specific gene predisposes to cancer. Two major genes involved in the pathogenesis of breast and ovarian cancer have been identified. *BRCA1* gene located on chromosome 17q21 [1, 2] and *BRCA2* gene located on chromosome 13q12 [3] are tumor suppressor genes involved in maintaining of genome integrity by engaging in many processes such as repair of DNA double strand breaks, cell cycle control and transcription [4]. Both genes explain a large part of families with a predisposition to breast and ovarian cancer [5]. Indeed, the risk of developing breast cancer in carriers of *BRCA1* or *BRCA2* mutation is about 45–80 % [6, 7].

Several studies have focused on clinical and pathological features of breast and ovarian cancer associated with *BRCA1* and *BRCA2* mutations [8–11]. These studies finding have shown that *BRCA1* and *BRCA2* tumors exhibit some specific morphological and immunohistochemical characteristics.

This study aims to compare the clinicopathological features between Moroccan breast cancers associated or not with *BRCA1* and *BRCA2* mutations in the order to find some clinical and pathological characteristics specific to this population especially that a recent study identified a specific founder *BRCA1* mutation in the Moroccan population [12].

## Methods

In our previous study [13], a total of 40 clinically high-risk breast and/or ovarian cancer patients, treated in Mohammed VI Cancer Treatment Center of Ibn Rochd University Hospital of Casablanca, were selected and referred for *BRCA* genetic testing to the Genetics and Molecular Pathology Laboratory of the Medical school of Casablanca between 2009 and 2010.

Breast cancer patients were selected according to specific criteria:

- Three or more first or second degree relatives with breast cancer diagnosed at any age in the same familial branch;
- Two first degree relatives with breast cancer, with at least one early onset breast cancer case (≤40 years) or male breast cancer case or ovarian cancer case.
- Single cases diagnosed with breast cancer before age 40.

As described previously [13], DNA was extracted from whole blood samples using the salting out method and all exons and exon–intron boundaries of *BRCA1* and *BRCA2* genes were amplified in a final volume of 25 μl containing: 1× reaction buffer, 1.5 mM MgCl2, 0.2 mM dNTPs, 5 μM primers (sequences available on request), 1.25 U Taq polymerase and 50 ng genomic DNA. Amplification cycles were: 94 °C for 7 min followed by 4 cycles of 94 °C for 0.1 min, 64 °C for 0.1 min, and 72 °C for 1 min, 4 cycles of 94 °C for 0.1 min, 64 °C for 0.1 min, and 72 °C for 1 min, 35 cycles of 94 °C for 0.1 min, 58 °C for 0.1 min, and 72 °C for 1 min and 1 cycle at 72 °C for 7 min, except for exon 15 of *BRCA2* for which the amplification conditions were: 94 °C for 7 min followed by 40 cycles of 94 °C for 1 min, 55 °C for 1 min, 72 °C for 2 min and ended with a 7 min incubation at 72 °C. Amplicons were purified and sequenced in both forward and reverse strands using BigDye Terminator v3.1 cycle sequencing kit (Applied Biosystems) then runned on a ABIPRISM 3130 XL Genetic analyzer (Applied Biosystems) after purification and denaturation. Sequence analyses were performed using SeqScape v2.6 (Applied Biosystems) software. All mutations and variants are cited according to Human Genome Variation Sequence systematic nomenclature (HGVS; http://www.hgvs.org/mutnomen/) using GenBank entries: U14680 for *BRCA1* and U43746 for *BRCA2*.

Sequencing results of the entire and exon/intron sequences of both genes have showed that 11 patients were mutated in *BRCA1*/*2* genes, and 29 women were not associated to *BRCA1*/*2* mutations.

A detailed semi-structured face to face interview including information on family history and risk factors for breast cancer (interview guide available upon request) such as age at diagnosis of breast cancer, age at menarche and menopause, parity (parous and nulliparous), breastfeeding (presence and absence), oral contraceptive use (presence and absence) and tumor location (unilateral or bilateral involvement) was conducted by HJ (a male professor of Radiation Oncology and PhD candidate with experience in conducting qualitative research) and AT (a female PhD researcher) at Mohammed VI Cancer Treatment Center of Ibn Rochd University Hospital of Casablanca after explaining the aim and the objectives of the study and obtaining written consent from all eligible women. All interviews were conducted in Moroccan Arabic language and lasted approximately 30 min. There were no third parties present for any interview. Collected data were recorded on transcripts which were not returned to interviewees then coded by both authors. None of the participants refused to participate and no repeat interviews were carried out. The histological analysis of the tumor, tumor size and lymph node involvement according to the TNM classification [14], SBR grade according to Nottingham modification of Scarff–Bloom–Richardson system [15], hormone receptor status and HER2 status based on CAP guidelines [16, 17] were collected by review of medical records.

The clinical and pathological features of patients carrying *BRCA1* or *BRCA2* mutation were evaluated and compared to those of non-mutated patients. Statistical analysis was performed using Epi Info Version 3.5.4. Quantitative variables with normal distribution were analyzed by Student’s t test. Comparison of qualitative data was performed using Fisher’s Exact test. The correlation is statistically significant between two variables if the P value is less than or equal to 0.05.

## Results

In this study, we tried to find a correlation between the clinicopathological characteristics of breast cancer and *BRCA1*/*2* mutation status. Indeed, our previous study [13] have revealed among 40 breast cancer patients, at increased risk of carrying a mutation, 29 women with negative *BRCA1*/*2* testing and 11 patients with a positive *BRCA1*/*2* status (Table 1) including six patients with *BRCA1* mutation and five patients carrying *BRCA2* mutation.

**Table 1:** *BRCA1* and *BRCA2* mutations [13]

Gene	Mutation	Manifestation, age at diagnosis	Family history
*BRCA1*	c.181T > G	BC, 34 years	Mother, BC 45 years
	c.798-799delTT	BC, 42 years	Mother, BOC 50 years
			M Cousin, BC 47 years
	c.2805delA	BC, 41 years	M aunt, BC 42 years
	c.3279delC	BC, 32 years	Mother, BC 49 years
	c.3279delC	BC, 49 years	Daughter, BC 32 years
	c.5062-5064delGTT	BC, 25 years	M aunt, CS 40 years
			M cousin, BC 27 years
*BRCA2*	c.745-1G > A	BC, 33 years	No family history
	c.3381delT	BC, 38 years	Mother, BC 50 years
	c.7110delA	BC, 38 years	M aunt, BC 43 years
			Mother, BC 40 years
	c.7235insG	BC, 40 years	M aunt, BC 38 years
			M aunt, BC 42 years
			M aunt, BC 45 years
			Sister, BC 33 years
	c.7235insG	BBC, 45 years	Sister, BC 41 years
			P cousin, BC 40 years
			P cousin, BC 50 years

*BC* breast cancer, *BBC* bilateral breast cancer, *BOC* breast and ovarian cancers, *M* maternal, *P* paternal

The main characteristics of breast cancer patients at diagnosis are shown in Table 2. In the present study, 90.9 % of *BRCA1*/*2* carriers versus 82.8 % of non carriers reported a family history (p = 0.66). The mean age at diagnosis of breast cancer and the mean age of first menarche was younger in *BRCA1*/*2* mutation carriers than in non-carriers (p = 0.05). Similarly, the average duration of breastfeeding was shorter among *BRCA1*/*2* carriers than non-carriers (p = 0.05). Conversely, no difference was observed between both groups regarding the use of oral contraceptives, age at first full-term pregnancy, parity, breastfeeding, age of menopause and tumor localization.

**Table 2:** Personal and clinical characteristics of patients carrying or not *BRCA1*/*2* mutations

Variable	*BRCA1*/*2*+	*BRCA1*/*2*−	p value
Family history			
No	1(9.1 %)	5 (17.2 %)	0.66
Yes	10 (90.9 %)	24 (82.8 %)	
Mean age at diagnosis (years)	37.90 (SD = 6.67)	44.48 (SD = 9.74)	*0.05*
Mean age at menarche (years)	13.08 (SD = 1.60)	14.24 (SD = 1.62)	*0.05*
Mean age at first delivery (years)	25.15 (SD = 5.76)	25.57 (SD = 6.60)	0.88
Mean age at menopause (years)	47.50 (SD = 0.70)	48.66 (SD = 5.60)	0.79
Breastfeeding			
No	4 (36.4 %)	12 (41.4 %)	1.00
Yes	7 (63.6 %)	17 (58.6 %)	
Average duration of breastfeeding (months)	8.71 (SD = 5.40)	19.35 (SD = 12.92)	*0.05*
Oral contraceptive use			
No	6 (54.5 %)	11 (37.9 %)	0.48
Yes	5 (45.5 %)	18 (62.1 %)	
Parity			
Nulliparous	4 (36.4 %)	10 (34.5 %)	1.00
Parous	7 (63.6 %)	19 (65.5 %)	
Tumor localization			
Unilateral	10 (90.9 %)	28 (96.6 %)	1.00
Bilateral	1 (9.1 %)	1 (3.4 %)	

Values in Italic are statistically significant (p value ≤ 0.05)

Histologically (Table 3), the infiltrating ductal carcinoma was the most common histological type in both groups (90.9 and 93.3 %). The medullary carcinoma accounted for 9.1 % in *BRCA1*/*2* carriers and only 3.4 % in non-carriers (not significant). T1 and T2 tumor sizes were observed in mutated patients with a frequency of 72.7 %. Moreover, SBR grade III was found in 63.6 % of women with *BRCA1*/*2* mutation against a frequency of 20.7 % among non-carriers, this difference appears to be statistically significant (p = 0.02). On the other hand, lymph node involvement, hormone receptors expression and Her2/neu status showed no statistically significant difference between both studied groups. However, *BRCA1*/*2* carriers were more likely to be triple-negative breast cancer compared with non-carriers (62.5 vs. 16.7 %, p = 0.02). Nevertheless, it should be emphasized that ER and PR status was not available in four patients (10 %) while Her2/neu data was missing in 8 (20 %). This may be due to the fact that some patients prefer to perform the tests in outside laboratories.

**Table 3:** Pathological characteristics of patients carrying or not *BRCA1/2* mutations

Variables	*BRCA1*/*2*+	*BRCA1*/2−	p value
Histological type			
Invasive ductal carcinoma	10 (90.9 %)	27 (93.1 %)	0.63
Invasive lobular carcinoma	0 (0 %)	1 (3.4 %)	
Medullary carcinoma	1 (9.1 %)	1 (3.4 %)	
Tumor size			
T1–T2	8 (72.7 %)	19 (65.5 %)	1.00
T3	2 (18.2 %)	(20.7 %)	
T4	1 (9.1 %)	4 (13.8 %)	
SBR grade			
I–II	4 (36.4 %)	23 (79.3 %)	*0.02*
III	7 (63.6 %)	6 (20.7 %)	
Node involvement			
N−	5 (45.5 %)	13 (44.8 %)	1.00
N+	6 (54.5 %)	16 (55.2 %)	
Estrogen receptors status			
ER−	7 (63.6 %)	10 (40.0 %)	0.28
ER+	4 (36.4 %)	15 (60.0 %)	
Progesterone receptors status			
PR−	7 (63.6 %)	11 (44.0 %)	0.47
PR+	4 (36.4 %)	14 (56.0 %)	
Her2/neu			
Her2−	5 (62.5 %)	16 (66.7 %)	1.00
Her2+	3 (37.5 %)	8 (33.3 %)	
Triple negative (ER−, PR− and HER2/neu−)			
Yes	5 (62.5 %)	4 (16.7 %)	*0.02*
No	3 (37.5 %)	20 (83.3 %)	

Values in Italic are statistically significant (p value ≤ 0.05)

## Discussion

Although the family history is widely established as a risk factor for breast cancer, there is a disagreement about its impact on prognosis with reported conflicting series results [18–25]. The discovery of *BRCA1* and *2* genes predisposing to breast cancer has improved identification of cases linked to genetic susceptibility.

The probability of an individual to carry a *BRCA1* or *BRCA2* germline mutation is based primarily on clinical data such as family history, age at diagnosis of breast cancer and ethnicity. Indeed, family history with a concentration of breast and ovarian cancers is the most important risk factor in developing the disease. However, this criterion presents some problems as it is based on the collection of the cancer events in the family without pathological confirmation. In addition, a number of population studies have revealed that a large proportion of breast cancer patients with a *BRCA1* or *BRCA2* germline mutation have no history of the disease in the family [26, 27].

Limited studies of *BRCA* gene mutations have been carried out in Morocco but none have described the clinicopathological characteristics in detail. Thus, the study of tumor phenotypes associated with *BRCA1*/*2* mutations may be useful to predict the probability to carry a germline mutation. In this study, we analyzed the clinicopathological characteristics of breast cancer patients based on their *BRCA* status.

Based on our results, the frequency of *BRCA1* and *BRCA2* mutations among Moroccan women with hereditary breast and/or ovarian cancer is 25.64 % [13]. Consistent with this result, a recent study [28] examining the prevalence of *BRCA1*/*2* germline mutations in 21,401 families with breast and ovarian cancer history has reported a prevalence of 24.0 % (95 % CI 23.4–24.6 %) [28].

Several studies have reported that breast cancer related to *BRCA1*/*2* mutations is often associated with an early age of diagnosis [10, 29–33]. Consistent with these findings, the comparison of the average age of diagnosis between our groups of breast cancer with or without *BRCA1*/*2* mutation showed a statistically significant difference. Semple et al. [34] recently reported that the annual breast cancer risks for *BRCA1* mutation is not affected by age of breast cancer diagnosis in the first-degree relative, which is not the case for *BRCA2* mutation carriers where women with a first-degree relative diagnosed before the age of 30 years have an annual breast cancer risk of 4.5 %.

On the other hand, the authors report that cumulative exposure to sex hormones, especially estrogen, is probably associated to breast cancer risk in *BRCA1* mutation carriers. Thus, women who had menarche at a later age or who have breastfed seem to be protected against breast cancer development, while the role of gender is ambiguous [35]. In this study, the age of the first menstruation seems to be statistically similar between *BRCA* mutation carriers and non-carriers. This observation was consistent with some previous studies [10, 30].

Our results showed that patients with or without *BRCA1*/*2* mutation were similar with regard to oral contraceptives use, age at first full-term pregnancy, parity, lactation and average age of menopause. These data are consistent with those reported in other investigations [8, 30]. However, the average duration of breastfeeding was statistically shorter in women carrying mutations. Jernström et al. [36] has observed a significantly shorter period of breastfeeding in *BRCA1* mutations women compared with non-carriers. In another study, cancer risk reductions were in the order of 32 and 49 % among women with *BRCA1* mutation who breastfed for at least one year (OR 0.68; 95 % CI 0.52–0.91; p = 0.008) and for two or more years (OR 0.51; 95 % CI 0.35–0.74; p = 0.0003), respectively. However, no significant association was observed between breastfeeding and breast cancer risk among *BRCA2* mutation carriers [35].

It is well known that breast cancer women with *BRCA1*/*2* mutation have a high risk of developing contralateral breast cancer. Indeed, a recent publication has reported a higher frequency of bilateral breast cancer in the *BRCA*-positive group [37]. However, Kwong et al. [30] had concluded that the bilateral nature of breast cancer was not significantly associated with *BRCA1* and *BRCA2* mutation which is in line with the results of the present study.

In general, a number of studies have raised some important biological and pathological differences between *BRCA1*/*2* mutations carriers and non-carriers. In our study, we observed a significant predominance of SBR grade III tumors (p = 0.02). Consistent with these findings, various reports have found that tumors related to *BRCA1*/*2* mutations seem to be of higher grade compared to non carriers [8, 30–32].

Additionally, tumor size and axillary dissection showed no statistically significant difference between both studied groups. In accordance with our results, Kwong et al. [30] had observed no difference in axillary lymph node involvement between breast cancers associated with *BRCA* mutations to those not related to mutations but they have found that *BRCA* carriers developed significantly smaller tumors [OR (T1 vs. T2–4) 0.41; 95 % CI 0.17–0.98; p = 0.05]. Contrariwise, another study had reported that *BRCA* positive patients tended to have positive lymphnodes [8].

Furthermore, our results showed no significant difference between both groups with regard to the expression of hormone and HER-2/neu receptors which is similar to the findings related to an Italian study [8]. Contrary to these results, some reports have found a significant difference between both groups regarding hormone and HER-2/neu receptors with a predominance of negative status in carriers of mutations [30, 38]. In this study, *BRCA1* and *BRCA2* carriers were more likely to have triple negative tumors (ER−, PR− and HER2/neu−) which is in line with literature [30–32, 39].

In considering the results of the present report, we should note that the sample size is very small witch means that the differences or similarities observed between *BRCA1*/*2* carriers and non carriers regarding the clinical and pathological characteristics studied maybe due to random variability. A limitation which may also be due to the fact that we restricted the study population to Moroccan women. Also, this study includes selected breast cancer patients with a high probability of carrying a pathogenic *BRCA1*/*2* germline mutation so it is not reasonable to generalize these results to the entire population. Therefore, these findings should be interpreted cautiously and need to be confirmed by larger trials.

## Conclusions

In this first Moroccan study comparing clinical and pathological characteristics of women carrying or not *BRCA* mutation, patients with *BRCA1*/*2* mutation tend to develop early breast cancer with high-grade tumors. On the other hand, early menarche and short duration of breastfeeding appear to characterize patients with *BRCA* mutation. Nevertheless, this study has a number of limitations; the main limitation is the reduced statistical power due to small sample size. Finally, further large scale research including more data is needed to better characterize the *BRCA1*/*2* cases and to evaluate the survival rate associated with these mutations in our population tumors. Moreover, it would be more interesting to study women with *BRCA1* and *BRCA2* mutations separately due to their differences in tumor characteristics.

## Abbreviations

BRCA1: breast cancer susceptibility gene 1
BRCA2: breast cancer susceptibility gene 2
SBR: Scarff–Bloom–Richardson
ER: estrogen receptor
PR: progesterone receptor
HER-2: human epidermal growth factor receptor 2

## References

[1] Hall JM, Lee MK, Newman B, Morrow JE, Anderson LA, Huey B, King MC (1990). Linkage of early-onset familial breast cancer to chromosome 17q21. Science.

[2] Miki Y, Swensen J, Shattuck-Eidens D, Futreal PA, Harshman K, Tavtigian S, Liu Q, Cochran C, Bennett LM, Ding W (1994). A strong candidate for the breast and ovarian cancer susceptibility gene BRCA1. Science.

[3] Wooster R, Neuhausen SL, Mangion J, Quirk Y, Ford D, Collins N, Nguyen K, Seal S, Tran T, Averill D (1994). Localization of a breast cancer susceptibility gene, BRCA2, to chromosome 13q12-13. Science.

[4] Yoshida K, Miki Y (2004). Role of BRCA1 and BRCA2 as regulators of DNA repair, transcription, and cell cycle in response to DNA damage. Cancer Sci.

[5] Ford D, Easton DF, Stratton M, Narod S, Goldgar D, Devilee P, Bishop DT, Weber B, Lenoir G, Chang-Claude J, Sobol H, Teare MD, Struewing J, Arason A, Scherneck S, Peto J, Rebbeck TR, Tonin P, Neuhausen S, Barkardottir R, Eyfjord J, Lynch H, Ponder BA, Gayther SA, Zelada-Hedman M (1998). Genetic heterogeneity and penetrance analysis of the BRCA1 and BRCA2 genes in breast cancer families. The Breast Cancer Linkage Consortium. Am J Hum Genet.

[6] King MC, Marks JH, Mandell JB, New York Breast Cancer Study Group (2003). Breast and ovarian cancer risks due to inherited mutations in BRCA1 and BRCA2. Science.

[7] Antoniou A, Pharoah PD, Narod S, Risch HA, Eyfjord JE, Hopper JL, Loman N, Olsson H, Johannsson O, Borg A, Pasini B, Radice P, Manoukian S, Eccles DM, Tang N, Olah E, Anton-Culver H, Warner E, Lubinski J, Gronwald J, Gorski B, Tulinius H, Thorlacius S, Eerola H, Nevanlinna H, Syrjäkoski K, Kallioniemi OP, Thompson D, Evans C, Peto J, Lalloo F, Evans DG, Easton DF (2003). Average risks of breast and ovarian cancer associated with BRCA1 or BRCA2 mutations detected in case series unselected for family history: a combined analysis of 22 studies. Am J Hum Genet.

[8] Veronesi A, de Giacomi C, Magri MD, Lombardi D, Zanetti M, Scuderi C, Dolcetti R, Viel A, Crivellari D, Bidoli E, Boiocchi M (2005). Familial breast cancer: characteristics and outcome of BRCA 1-2 positive and negative cases. BMC Cancer.

[9] Musolino A, Bella MA, Bortesi B, Michiara M, Naldi N, Zanelli P, Capelletti M, Pezzuolo D, Camisa R, Savi M, Neri TM, Ardizzoni A (2007). BRCA mutations, molecular markers, and clinical variables in early-onset breast cancer: a population-based study. Breast.

[10] Atchley DP, Albarracin CT, Lopez A, Valero V, Amos CI, Gonzalez-Angulo AM, Hortobagyi GN, Arun BK (2008). Clinical and pathologic characteristics of patients with BRCA-positive and BRCA-negative breast cancer. J Clin Oncol.

[11] Tung N, Miron A, Schnitt SJ, Gautam S, Fetten K, Kaplan J, Yassin Y, Buraimoh A, Kim JY, Szász AM, Tian R, Wang ZC, Collins LC, Brock J, Krag K, Legare RD, Sgroi D, Ryan PD, Silver DP, Garber JE, Richardson AL (2010). Prevalence and predictors of loss of wild type BRCA1 in estrogen receptor positive and negative BRCA1-associated breast cancers. Breast Cancer Res.

[12] Quiles F, Teulé À, Martinussen Tandstad N, Feliubadaló L, Tornero E, Del Valle J, Menéndez M, Salinas M, Wethe Rognlien V, Velasco A, Izquierdo A, Capellá G, Brunet J, Lázaro C (2016). Identification of a founder BRCA1 mutation in the Moroccan population. Clin Genet.

[13] Tazzite A, Jouhadi H, Nadifi S, Aretini P, Falaschi E, Collavoli A, Benider A, Caligo MA (2012). BRCA1 and BRCA2 germline mutations in Moroccan breast/ovarian cancer families: novel mutations and unclassified variants. Gynecol Oncol.

[14] Edge SB, Byrd DR, Compton CC, Fritz AG, Greene FL, Trotti A (2010). AJCC cancer staging manual.

[15] Elston CW, Ellis IO (1991). Pathological prognostic factors in breast cancer. I. The value of histological grade in breast cancer: experience from a large study with long-term follow-up. Histopathology.

[16] Wolff AC, Hammond ME, Schwartz JN, Hagerty KL, Allred DC, Cote RJ, Dowsett M, Fitzgibbons PL, Hanna WM, Langer A, McShane LM, Paik S, Pegram MD, Perez EA, Press MF, Rhodes A, Sturgeon C, Taube SE, Tubbs R, Vance GH, van de Vijver M, Wheeler TM, Hayes DF (2007). American Society of Clinical Oncology/College of American Pathologists guideline recommendations for human epidermal growth factor receptor 2 testing in breast cancer. J Clin Oncol.

[17] Hammond ME, Hayes DF, Dowsett M, Allred DC, Hagerty KL, Badve S, Fitzgibbons PL, Francis G, Goldstein NS, Hayes M, Hicks DG, Lester S, Love R, Mangu PB, McShane L, Miller K, Osborne CK, Paik S, Perlmutter J, Rhodes A, Sasano H, Schwartz JN, Sweep FC, Taube S, Torlakovic EE, Valenstein P, Viale G, Visscher D, Wheeler T, Williams RB, Wittliff JL, Wolff AC (2010). American Society of Clinical Oncology/College of American Pathologists guideline recommendations for immunohistochemical testing of Estrogen and Progesterone Receptors in Beast Cancer. J Clin Oncol.

[18] Albano WA, Recabaren JA, Lynch HT, Campbell AS, Mailliard JA, Organ CH, Lynch JF, Kimberling WJ (1982). Natural history of hereditary cancer of the breast and colon. Cancer.

[19] Andersson M, Daugaard S, von der Maase H, Mouridsen HT (1986). Doxorubicin versus mitomycin versus doxorubicin plus mitomycin in advanced breast cancer: a randomized study. Cancer Treat Rep.

[20] Fukutomi T, Kobayashi Y, Nanasawa T, Yamamoto H, Tsuda H (1993). A clinicopathological analysis of breast cancer in patients with a family history. Surg Today.

[21] Slattery ML, Berry TD, Kerber RA (1993). Is survival among women diagnosed with breast cancer influenced by family history of breast cancer?. Epidemiology.

[22] Chen LM, Mundt AJ, Power C, Halpern HJ, Weichselbaum RR (1996). Significance of family history in breast cancer treated with breast conservation therapy. Breast J.

[23] Russo A, Herd-Smith A, Gestri D, Bianchi S, Vezzosi V, Rosselli Del Turco M, Cardona G (2002). Does family history influence survival in breast cancer cases?. Int J Cancer.

[24] Thalib L, Wedrén S, Granath F, Adami HO, Rydh B, Magnusson C, Hall P (2004). Breast cancer prognosis in relation to family history of breast and ovarian cancer. Br J Cancer.

[25] Chang ET, Milne RL, Phillips KA, Figueiredo JC, Sangaramoorthy M, Keegan TH, Andrulis IL, Hopper JL, Goodwin PJ, O’Malley FP, Weerasooriya N, Apicella C, Southey MC, Friedlander ML, Giles GG, Whittemore AS, West DW, John EM (2009). Family history of breast cancer and all-cause mortality after breast cancer diagnosis in the Breast Cancer Family Registry. Breast Cancer Res Treat.

[26] Hopper JL, Chenevix-Trench G, Jolley DJ, Dite GS, Jenkins MA, Venter DJ, McCredie MR, Giles GG (1999). Design and analysis issues in a population-based, case–control-family study of the genetic epidemiology of breast cancer and the Co-operative Family Registry for Breast Cancer Studies (CFRBCS). J Natl Cancer Inst Monogr..

[27] Peto J, Collins N, Barfoot R, Seal S, Warren W, Rahman N, Easton DF, Evans C, Deacon J, Stratton MR (1999). Prevalence of BRCA1 and BRCA2 gene mutations in patients with early-onset breast cancer. J Natl Cancer Inst.

[28] Kast K, Rhiem K, Wappenschmidt B, Hahnen E, Hauke J, Bluemcke B, Zarghooni V, Herold N, Ditsch N, Kiechle M, Braun M, Fischer C, Dikow N, Schott S, Rahner N, Niederacher D, Fehm T, Gehrig A, Mueller-Reible C, Arnold N, Maass N, Borck G, de Gregorio N, Scholz C, Auber B, Varon-Manteeva R, Speiser D, Horvath J, Lichey N, Wimberger P, Stark S, Faust U, Weber BH, Emons G, Zachariae S, Meindl A, Schmutzler RK, Engel C, German Consortium for Hereditary Breast and Ovarian Cancer (GC-HBOC) (2016). Prevalence of BRCA1/2 germline mutations in 21,401 families with breast and ovarian cancer. J Med Genet.

[29] Li WF, Hu Z, Rao NY, Song CG, Zhang B, Cao MZ, Su FX, Wang YS, He PQ, Di GH, Shen KW, Wu J, Lu JS, Luo JM, Liu XY, Zhou J, Wang L, Zhao L, Liu YB, Yuan WT, Yang L, Shen ZZ, Huang W, Shao ZM (2008). The prevalence of BRCA1 and BRCA2 germline mutations in high-risk breast cancer patients of Chinese Han nationality: two recurrent mutations were identified. Breast Cancer Res Treat.

[30] Kwong A, Wong LP, Wong HN, Law FB, Ng EK, Tang YH, Chan WK, Suen DT, Choi C, Ho LS, Kwan KH, Poon M, Wong TT, Chan K, Chan SW, Ying MW, Chan WC, Ma ES, Ford JM, West DW (2009). Clinical and pathological characteristics of Chinese patients with BRCA related breast cancer. Hugo J.

[31] Chen W, Pan K, Ouyang T, Li J, Wang T, Fan Z, Fan T, Lin B, Lu Y, You W, Xie Y (2009). BRCA1 germline mutations and tumor characteristics in Chinese women with familial or early-onset breast cancer. Breast Cancer Res Treat.

[32] Zhang J, Pei R, Pang Z, Ouyang T, Li J, Wang T, Fan Z, Fan T, Lin B, Xie Y (2012). Prevalence and characterization of BRCA1 and BRCA2 germline mutations in Chinese women with familial breast cancer. Breast Cancer Res Treat.

[33] Cao W, Wang X, Gao Y, Yang H, Li JC (2013). BRCA1 germ-line mutations and tumor characteristics in eastern Chinese women with familial breast cancer. Anat Rec (Hoboken).

[34] Semple J, Metcalfe KA, Lubinski J, Huzarski T, Gronwald J, Armel S, Lynch HT, Karlan B, Foulkes W, Singer CF, Neuhausen SL, Eng C, Iqbal J, Narod SA, Hereditary Breast Cancer Clinical Study Group (2015). Does the age of breast cancer diagnosis in first-degree relatives impact on the risk of breast cancer in BRCA1 and BRCA2 mutation carriers?. Breast Cancer Res Treat.

[35] Kotsopoulos J, Lubinski J, Salmena L, Lynch HT, Kim-Sing C, Foulkes WD, Ghadirian P, Neuhausen SL, Demsky R, Tung N, Ainsworth P, Senter L, Eisen A, Eng C, Singer C, Ginsburg O, Blum J, Huzarski T, Poll A, Sun P, Narod SA, Hereditary Breast Cancer Clinical Study Group (2012). Breastfeeding and the risk of breast cancer in BRCA1 and BRCA2 mutation carriers. Breast Cancer Res.

[36] Jernström H, Johannsson O, Borg Å, Olsson H (1998). Do BRCA1 mutations affect the ability to breast-feed? Significantly shorter length of breast-feeding among BRCA1 mutation carriers compared with their unaffected relatives. Breast.

[37] Noh JM, Choi DH, Baek H, Nam SJ, Lee JE, Kim JW, Ki CS, Park W, Huh SJ (2012). Associations between BRCA mutations in high-risk breast cancer patients and familial cancers other than breast or ovary. J Breast Cancer.

[38] Yip CH, Taib NA, Choo WY, Rampal S, Thong MK, Teo SH (2009). Clinical and pathologic differences between BRCA1-, BRCA2-, and non-BRCA-associated breast cancers in a multiracial developing country. World J Surg.

[39] Bayraktar S, Gutierrez-Barrera AM, Liu D, Tasbas T, Akar U, Litton JK, Lin E, Albarracin CT, Meric-Bernstam F, Gonzalez-Angulo AM, Hortobagyi GN, Arun BK (2011). Outcome of triple-negative breast cancer in patients with or without deleterious BRCA mutations. Breast Cancer Res Treat.

